# Thoughts on Physical Education, and the True Method of Improving the Condition of Man

**Published:** 1837-04

**Authors:** 


Art. IV.—*Thoughts on Physical Education, and the true Method of Improving the Condition of Man.* By Charles Caldwell, m.d., Professor of the Institutes of Medicine and Clinical Practice in Transylvania University. *With Notes*, by Robert Cox; *and a recommendatory Preface*, by George Combe.—*Edinburgh*, 1836. 12mo. pp. 190.

This, like some other treatises we have had the pleasure of reading from the pen of Dr. Caldwell, is characterized by originality of thought and expression; it strikes at the root of existing prejudices, and is replete with truths which, if universally diffused and acted upon, would, without doubt, consummate the author's wishes. For its style, the logical accuracy pervading it, but much more for the immense mass of information connected with the leading principles of physical education, it is well worthy a place by the side of the popular writings of Dr. Combe.

The following passage shows in how comprehensive a sense the subject of his treatise is regarded by the author:

"By education in the abstract, I mean *a scheme of action or training, by which any form of living matter may be improved, and, by perseverance, reared to the highest perfection of which it is susceptible.* I say 'any form;' because the lower orders of living beings, vegetables not excepted, may be educated and improved, as certainly as the higher, and on the same grounds. That it may produce the desired effect, the scheme pursued must conform to the constitution of the race of beings for whose improvement it is intended; and, in the present instance, that race is our own." (P. 6.)

Dr. Caldwell answers in a very triumphant manner that class of philosophers who assert that education can, and does, improve the abstract mind, but that no corresponding change or amendment takes place in the instruments with which it works.

"The organized system of man," says the author, "constitutes the machinery with which alone his mind operates during their connexion as soul and body. Improve the apparatus, then, and you facilitate and improve the work which the mind performs with it, precisely as you facilitate steam operation, and enhance its product, by improving the machinery with which it is executed. In one case steam, and in the other spirit, continue unchanged; and each works and produces with a degree of perfection corresponding to that of the instruments it employs." (P. 7.)

When we speak of the superiority of any particular sense, as vision, this superiority is universally referred to an improved condition of the apparatus by which the eye is enabled to perform its functions. The effect produced does not depend upon an alteration in abstract mind, but upon some important amendment in organized matter. Such being the fact as regards the external senses, the author asks for the same admission respecting the higher mental operations.

"In performing them, the mind works with the brain as its machinery, as certainly as it does with the eye in seeing, or the muscles in dancing and swordmanship. Is any form of memory—say the memory of words, or that of places—rendered more apt and retentive by judicious exercise? We have no reason to believe that the mind or spirit is amended in this instance, any more than in those heretofore enumerated. It is a portion of the brain, the organ of language or locality, that is amended. By practice, man becomes more powerful and adroit in reasoning and judging. Here, again, the mind is not changed. The belief to that effect has no shadow of evidence to sustain it. The improvement in this case, as in the preceding ones, is confined to the organs with which the mind reasons and judges." (P. 8.)

It will be perceived that Dr. Caldwell's doctrines are based on phrenology, of which he appears to be a zealous cultivator. We are far from quarrelling with these so long as they are restricted to the ascertained facts of this new science, which is certainly calculated to do more for education than all preceding systems. Dr. Caldwell justly finds fault with those writers upon education who treat technically of moral, intellectual, and physical education; and suggests that they should rather "speak correctly of the education of the different portions of the body, each portion being trained according to its organization and character."

"The skin, for example, must be educated by one mode of discipline, the stomach by another, the lungs by a third, the muscles and circulatory system by a fourth, and each external sense and cerebral organ by a method corresponding to the peculiarity of its nature. In this view of the subject, which is the only rational one, the training of the brain in all its departments, by whatever name they may be called, is as truly a *physical or physiological process* as the training of any other part of the body." (P. 13.)

Dr. Caldwell dwells on the paramount importance of directing attention to the fact, that constitutional qualities are transmitted from parents to their children, and shews the reason why children born at the different periods of matrimonial alliance differ so materially, both as regards mental and bodily power. This is a subject apparently new, or at any rate but little attended to. Common observation proves that a great difference exists in a family of children, more especially as regards mental and bodily aptitude; and this, it is supposed, must arise from the state of health of the parents at the time of its production, and much more from the degree of maturity which they have attained. Any difference in education is insufficient to account for it, because it is exhibited in a minor degree, long before this can have had much influence. Our author thus explains the fact:

"Very young parents are, in constitution, immature and comparatively feeble; and that constitutional imperfection descends to their early offspring. As years pass on, their being ripens, and their strength increases. As a natural effect of this, the constitutions of their children become ameliorated. For reasons well known to phrenologists, the animal organs and faculties predominate during early life. Parents, therefore, who marry at that period communicate in a higher degree to their first children the same unfortunate predominance, which renders them less intellectual and moral, and more sensual; less capable, as well as less ambitious of preeminence in knowledge and virtue, and more inclined to animal indulgences. If I am not mistaken, history and observation sustain this view of the subject, and philosophy expounds it." (P. 16.)

Dr. C. very properly and forcibly insists that early marriages are materially concerned in retarding the improvement of our race, and on this subject thus expresses himself:—

"When a skilful agriculturist wishes to amend his breed of cattle, he does not employ for that purpose immature animals. On the contrary, he carefully prevents their intercourse. Experience, moreover, teaches him not to expect fruit of the best quality from immature fruit trees or vines. The product of such crudeness is always defective. In like manner, marriages between boarding-school girls and striplings in or just out of college, ought to be prohibited. In such cases, prohibition is a duty, no less to the parties themselves than to their offspring and society. Marriages of this kind are rarely productive of any thing desirable. Mischief and unhappiness of some sort are their natural fruit. Patriotism, therefore, philanthropy, and every feeling of kindness to human nature, call for their prevention." (P. 18.)

The following remarks on the injudicious method adopted by some parents and instructors of youth, of stimulating the yet immature brain of the child by studies, which ought not to be entered upon for some years, are deserving of the most serious attention; and, although they are, in some degree, a repetition of what we have already said in some of our previous Numbers, they can never be deemed superfluous:

"When the business of education shall be thoroughly understood, the brain of infants will be then no longer neglected as a mass of matter of little importance: skin, muscle, and bone being thought preferable to it. On the contrary, it will be viewed in its true character, as the ruling organ of the body, and the apparatus of the mind and its training will receive the attention it merits. I repeat,—and the repetition should be persevered in until its truth be acknowledged and reduced to practice,—that most of the evils of education under which the world has so long suffered, and is still suffering, arise from the mistaken belief that, in what is called moral and intellectual education, it is the *mind* that is exercised, and not the *brain*.

"No part is so easily or ruinously deranged by excessive action as the brain, especially the half-formed and highly susceptible brain of infants. Let this truth be realized, and faithfully and skilfully acted on, and human suffering from hydrocephalus, rickets, phrenitis, idiocy, epilepsy, madness, and other cerebral affections will be greatly diminished." (P. 39.)

If there be any one part of the system of education in which the writings of Drs. Caldwell, Combe, and Brigham will be of more peculiar and powerful service, it is in pointing out the effects produced by over-stimulating the infant brain; a condition which produces, in the first place, disease of that organ, and deranges secondarily the functions of others. What is it but ignorance of the laws affecting organized matter, and the knowledge that the mind acts through its medium, which prevents parents, friends, and tutors from seeing the cause of the dreadful results which overtake their infant prodigies? The instructors themselves know full well the enfeebling and destructive tendency which an overloaded and over-excited stomach exerts upon their digestion; but, although the same law is in operation, they seem not to be aware that, by over-exciting the brain, by imposing upon it work while it is yet undeveloped, they as certainly enfeeble and destroy the mind. It is to writers on phrenology chiefly that we are indebted for calling attention to the fact that the cerebral organs might be fatigued by too much exercise, brought into a state of considerable irritation, and that inflammation might follow, and all the dire results attending this process: that, although the injurious effect might be slow in following its cause, the foundation might be laid for various species of insanity. An aptitude for disease is thus produced in the cerebral organs, and the law of inheritance teaches us that the effect will not die with the individual subjected to the erroneous training, but that this individual's children may resemble him, and become in their turn the subjects of mental aberration.

The following remarks also particularly merit notice: we are happy to think that they have already acquired it in several well-conducted schools. The facts are certain, whatever our readers may be disposed to think of the explanation.

"By changing from one study to another successively in the same day, those who are cultivating science and letters not only learn much more than they could under confinement to a single study, but do so with less exhaustion and danger to health. Why? Because, by closely studying one branch of knowledge only,—in other words, by labouring all day with one cerebral organ,—it becomes exhausted and dull, as every industrious student must have felt. When thus worn out, therefore, by toil, not only is it unfit to exercise further with due effect, and master its task, but its health is endangered, if not for the time actually injured. It is in a fatigued condition, which borders on a diseased one, and often excites it. When, on the contrary, the pupil, feeling himself becoming unfit for one study, passes to another, he engages in the latter with a fresh and active organ and makes rapid progress in it, until, beginning to be again fatigued and dull, he changes to a third, or returns to that previously relinquished, the organ corresponding to it being reinvigorated by rest." (P. 68.)

Much interesting matter follows on the subjects of Insanity and Dyspepsia, indicating how frequently these diseases are combined in the same individual, and illustrating the important practical fact, which cannot be too strongly impressed on the minds of physicians, that cerebral irritation is one of the most frequent causes of dyspepsia.

We conclude by strongly recommending this little work to our readers, especially such as are themselves parents.

